# Non-canonical Activities of Hog1 Control Sensitivity of *Candida albicans* to Killer Toxins From *Debaryomyces hansenii*

**DOI:** 10.3389/fcimb.2018.00135

**Published:** 2018-05-03

**Authors:** Ana Morales-Menchén, Federico Navarro-García, José P. Guirao-Abad, Elvira Román, Daniel Prieto, Ioana V. Coman, Jesús Pla, Rebeca Alonso-Monge

**Affiliations:** Departamento de Microbiología y Parasitología, Facultad de Farmacia, Universidad Complutense de Madrid, Madrid, Spain

**Keywords:** killer toxin, osmotic stress, *Debaryomyces hansenii*, MAPK, *Candida albicans*, HOG pathway

## Abstract

Certain yeasts secrete peptides known as killer toxins or mycocins with a deleterious effect on sensitive yeasts or filamentous fungi, a common phenomenon in environmental species. In a recent work, different *Debaryomyces hansenii* (*Dh*) strains isolated from a wide variety of cheeses were identified as producing killer toxins active against *Candida albicans* and *Candida tropicalis*. We have analyzed the killer activity of these toxins in *C. albicans* mutants defective in MAPK signaling pathways and found that the lack of the MAPK Hog1 (but not Cek1 or Mkc1) renders cells hypersensitive to *Dh* mycocins while mutants lacking other upstream elements of the pathway behave as the wild type strain. Point mutations in the phosphorylation site (T174A-176F) or in the kinase domain (K52R) of *HOG1* gene showed that both activities were relevant for the survival of *C. albicans* to *Dh* killer toxins. Moreover, Hog1 phosphorylation was also required to sense and adapt to osmotic and oxidative stress while the kinase activity was somehow dispensable. Although the addition of supernatant from the killer toxin- producing *D. hansenii* 242 strain (*Dh*-242) induced a slight intracellular increase in Reactive Oxygen Species (ROS), overexpression of cytosolic catalase did not protect *C. albicans* against this mycocin. This supernatant induced an increase in intracellular glycerol concentration suggesting that this toxin triggers an osmotic stress. We also provide evidence of a correlation between sensitivity to *Dh*-242 killer toxin and resistance to Congo red, suggesting cell wall specific alterations in sensitive strains.

## Introduction

Killer toxins or mycocins are yeast (glyco)proteins with a toxic effect against other sensitive yeasts. They were first described in *Saccharomyces cerevisiae* in 1963 and are grouped in three types (K1, K2, and K28) according to the set of sensitive strains and lack of cross-immunity as killer toxin-producing yeasts are resistant to their own toxins (Marquina et al., 2002). More recently, a new killer toxin from wine maker *S. cerevisiae* strains, named Klus, was reported to kill all other mycocin producing *S. cerevisiae* strains (Rodríguez-Cousiño et al., 2011). Killer toxins are not exclusive to *S. cerevisiae* and other yeast genera such as *Candida, Cryptococcus, Debaryomyces, Hansenula, Pichia*, or *Kluyveromyces* have been reported to produce them. Killer toxins are protease-sensitive proteins, most of them only stable and active at acidic pH and low temperatures, losing activity above 35°C and a pH above 6. Nevertheless, some killer toxins are stable at a wide range of pH and high temperature like HM-1 from *Hansenula makrii* which is stable at pH 2–11 and 60°C for 1 h. Many of the genes that encode these killer toxins have been identified and their mechanism of action elucidated (Liu et al., 2015). K1 from *S. cerevisiae* (Breinig et al., 2002) and PMKT from *Pichia membranifaciens* (Santos and Marquina, 2004) induce membrane pore formation while K28 from *S. cerevisiae* and PMKT2 from *P. membranifaciens* arrest cell cycle at an early S phase (Schmitt et al., 1996; Santos et al., 2013). PMKT2 is also able to induce apoptosis in sensitive yeast (Santos et al., 2013). *Kluyveromyces lactis* produces a zymocin with tRNase activity (Jablonowski and Schaffrath, 2007) and *Hansenula mrakii* HM-1 inhibits the beta-1,3-glucan synthase activity thus, perturbing bud formation and conjugation in yeasts (Takasuka et al., 1995).

Mycocin-producing yeasts are ubiquitous in natural habitats, and can be isolated from soil, water or plants as well as in several foods and beverages. A potential application of these killer toxins could be the control of yeast populations during wine or beer fermentation, the inhibition of fungi contaminating food or fungal pathogens in plants (Schmitt and Breinig, 2002; Breinig et al., 2006). Moreover, a potential use in human and animal health has also been proposed as many killer toxins have been reported to have activity against *C. albicans* (Magliani et al., 1997; Polonelli et al., 2011), the main opportunistic fungal pathogen in humans and the fourth cause of nosocomial systemic infections in many developed countries (Pfaller and Diekema, 2007, 2010). This pathogen forms part of the microbiota in healthy individuals as a harmless commensal, causing diseases only when the immune system or the natural barriers become altered. Many efforts have been made to understand the virulence factors involved in the commensal to pathogen transition in *C. albicans* (Tang et al., 2016). Among others, the ability to adapt to different niches has been proposed to play a relevant role in *C. albicans* pathogenicity (Recently reviewed by Mayer et al., 2013; Poulain, 2015; Höfs et al., 2016). The ability to sense and respond to environmental changes allowing adaptation and survival is mediated by conserved signal transduction pathways. MAP Kinase pathways are some of the most studied signaling mechanisms in *C. albicans* (Alonso-Monge et al., 2006; Román et al., 2007). These MAPK kinase pathways integrate inputs and coordinate responses that allow the survival and adaptation to different environmental conditions. These pathways are integrated by transmembrane proteins, sensors, intermediate proteins and a MAPK module that includes three protein kinases that become sequentially activated by phosphorylation. The HOG pathway has been reported to be activated in response to several stresses and plays a role in cell wall biosynthesis, morphological transitions, tolerance to phagocytes, virulence and adaptation to commensalism (Alonso-Monge et al., 1999; Arana et al., 2007; Prieto et al., 2014). In *S. cerevisiae*, the phosphorylation of the Hog1 homolog was reported to be triggered by the killer toxin from *P. membranifaciens* PMKT (Santos et al., 2005). More recently Banjara and co-workers identified 23 *Debaryomyces hansenii* strains that exerted killer effects over *C. albicans* and *C. tropicalis*. These *D. hansenii* strains were isolated from cheeses of different origins and their toxins displayed different activities depending on the strain, the temperature and pH, thus opening the possibility to control *C. albicans* infections through a new therapeutic approach (Banjara et al., 2016).

In the present study, the killer activity of seven of the *D. hansenii* mycocin-producing strains from Banjara's work was tested against signal transduction *C. albicans* mutants in order to understand their mechanism of action. We show how these toxins have an increased action on specific MAPK mutants (*hog1*) but not others (*cek1* or *mkc1*) suggesting a role for the HOG MAPK in killer toxin resistance in *C. albicans*. We also characterize their effect on cellular physiology and the biochemical role of the Hog1 MAP kinase in this process.

## Materials and methods

### Strains and growth conditions

*Candida albicans* strains used in the present work are listed in Table 1. *D. hansenii* strains and their origin are listed in Table 2 (Banjara et al., 2016). *D. hansenii* strains were routinely incubated at 30°C while *C. albicans* strains at 37°C. Yeasts were grown in YPD medium (1% yeast extract, 2% peptone, 2% glucose). Usually, overnight cultures were used for sensitivity assays. For this purpose, cells were suspended to an O.D._600nm_ = 0.8 and 5 μL of 10 fold serial dilutions were plated on YPD solid media supplemented with the different compounds at their indicated concentrations.

**Table 1:** *C. albicans* strains used in this work.

**Microorganism**	**Strain**	**Genotype**	**Nomenclature in manuscript and figures**	**References**
*C. albicans*	*SC5314*	*wt*		Gillum et al., 1984
*C. albicans*	*CAF2-1*	*ura3::imm434/ura3::imm434-URA3*	*CAF2 (wt)*	Fonzi et al., 1993
*C. albicans*	*CHO4-1*	*ura3::imm434/ura3::imm434-URA3 opy2::FRT/opy2::FRT*	*opy2*	Herrero de Dios, 2013
*C. albicans*	*CK43B-16*	*ura3::imm434/ura3::imm434 cek1::hisG/cek1::hisG-URA3-hisG*	*cek1*	Csank et al., 1998
*C. albicans*	*CSSK21-U-6*	*URA3/ura3Δ::imm434 ssk1::hisG/ssk1::hisG-URA3-hisG*	*ssk1*	Calera et al., 2000
*C. albicans*	*CM1613*	*ura3Δ::imm434/ura3Δ::imm434 mkc1::hisG/mkc1::hisG–URA3–hisG*	*mkc1*	Navarro-García et al., 1995
*C. albicans*	*HI3-21*	*ura3Δ::imm434/ura3Δ::imm434 hog1::hisG-URA3-hisG/hog1::hisG*	*hog1*	Prieto et al., 2014
*C. albicans*	*RM100*	*ura3::imm434/ura3::imm434 his1::hisG/his1::hisG-URA3-hisG*	*RM100 (wt)*	Negredo et al., 1997
*C. albicans*	*CNC13*	*RM1000 hog1::hisG-URA3-hisG/hog1::hisG*	*hog1 (RM100)*	San José et al., 1996
*C. albicans*	*BRD3*	*RM1000 pbs2Δ::cat/pbs2Δ::cat-URA3-cat*	*pbs2*	Arana et al., 2005
*C. albicans*	*BRD7*	*RM1000 hog1::hisG/hog1::hisG pbs2Δ::cat/pbs2Δ::cat-URA3-cat*	*hog1 pbs2*	Arana et al., 2005
*C. albicans*	*VIC100*	*ura3Δ::imm434/ura3Δ::imm434 his1Δ::hisG/his1Δ::hisG sko1Δ::hisG/sko1Δ::hisG-URA3-hisG*	*sko1*	Alonso-Monge et al., 2010
*C. albicans*	*VIC200*	*ura3Δ::imm434/ura3Δ::imm434 his1Δ::hisG/his1Δ::hisG hog1::hisG/hog1::hisG sko1Δ::hisG/sko1Δ::hisG-URA3-hisG*	*hog1 sko1*	Alonso-Monge et al., 2010
*C. albicans*	*REP3*	*ura3::imm434/ura3::imm434 his1::hisG/his1::hisG sho1::hisG/sho1::hisG-URA3-hisG*	*sho1*	Román et al., 2005
*C. albicans*	*AMB4*	*URA3/ura3Δ::imm434 mkk2Δ::FTR/mkk2Δ::FTR*	*mkk2*	Román et al., 2015
*C. albicans*	*REP18-1*	*ura3::imm434/ura3::imm434 his1::hisG/his1::hisG-URA3-hisG msb2::FRT/msb2::FRT*	*msb2*	Román et al., 2009
*C. albicans*	*COA6*	*ura3::imm434/ura3::imm434 ADH1/adh1::tTA pTet-GFP-SAT1*	*CAF2-pNIM1R*	Prieto et al., 2014
*C. albicans*	*HHH*	*ura3Δ::imm434/ura3Δ::imm434 hog1::hisG-URA3-hisG/hog1::hisG ADH1/adh1::tTA Ptet–HOG1-SAT1*	*hog1-pNIM1R-HOG1*	This work
*C. albicans*	*HHA*	*ura3Δ::imm434/ura3Δ::imm434 hog1::hisG-URA3-hisG/hog1::hisG ADH1/adh1::tTA Ptet–hog1^*F*321*L*^-SAT1*	*hog1^*F*321*L*^*	This work
*C. albicans*	*HKD*	*ura3Δ::imm434/ura3Δ::imm434 hog1::hisG-URA3-hisG/hog1::hisG ADH1/adh1::tTA Ptet–hog1^*K*52*RL*^-SAT1*	*hog1^*K*52*R*^*	This work
*C. albicans*	*HSP*	*ura3Δ::imm434/ura3Δ::imm434 hog1::hisG-URA3-hisG/hog1::hisG ADH1/adh1::tTA Ptet–hog1^*T*174*A*^-SAT1*	*hog1^*T*174*A*^*	This work
*C. albicans*	*HDP*	*ura3Δ::imm434/ura3Δ::imm434 hog1::hisG-URA3-hisG/hog1::hisG ADH1/adh1::tTA Ptet–hog1^*T*174*A*−*Y*176*F*^-SAT1*	*hog1 ^*T*174*A*−*Y*176*F*^*-	This work
*C. albicans*	*PPD7*	ura3Δ::imm434/URA3 ADH1/adh1::tTA P_*tet*_ -dTOM2-SAT1	*CAI4-dTOM2*	Prieto et al., 2014
*C. albicans*	*HRFP*	*ura3Δ::imm434/ura3Δ::imm434 hog1::hisG-URA3-hisG/hog1::hisG* ADH1/adh1::tTA P_*tet*_ -dTOM2-SAT	*hog1-dTOM2*	This work
*C. albicans*	*REP41*	*ura3Δ::imm434/ura3Δ::imm434 hog1::hisG- /hog1::hisG ADH1/adh1::tTA-URA3*	*hog1-pNRU-e*	This work
*C. albicans*	*REP32*	*ura3Δ::imm434/ura3Δ::imm434 hog1::hisG- /hog1::hisG ADH1/adh1::tTA Ptet–CAT1-URA3*	*hog1-pNRU-CAT1*	This work

**Table 2:** Killer toxin-producing strains used in this work.

	**Microorganism**	**Origin**
*Dh*-65	*Debaryomyces hansenii*	Gouda cheese isolate (Netherlands)
*Dh*-72	*D. hansenii*	Colby cheese isolate (Wisconsin. US)
*Dh*-220	*D. hansenii*	Bel Paese cheese isolate (Italy)
*Dh*-242	*D. hansenii*	Parmesan cheese isolate (Parma. Italy)
*Dh*-246	*D. hansenii*	Raclette cheese isolate (Wisconsin. US)
*Dh*-262	*D. hansenii*	Ricotta cheese isolate (Illinois. US)
*Dh*-274	*D. hansenii*	Blue 2 cheese isolate (Wisconsin. US)
CBS-767	*Candida boidinii IGC3430*	

### Molecular biology procedures and plasmid constructions

The *HOG1* gene inserted in a pUC19 vector was mutated using a commercial kit (QuickChange, Stratagene) and specific primers (Table 3). *HOG1* mutated versions were amplified using the primers up_HOG_myc (GCCTCGAGATGTCTGCAGATGGAGAATTTACAAGA) and Low_HOG_myc (CTGCGGCCGCTAGCTCCGTTGGCGAATCC), digested with *Sal*I-*Not*I and integrated in pNIM1R-RFP (Prieto et al., 2014) replacing the RFP gene. The generated plasmids pNIM1R-HOG1#-myc were digested with *Kpn*I-*Sac*II and integrated into the *ADH1 locus* of *C. albicans* genome using lithium acetate transformation (Köhler et al., 1997).

**Table 3:** Primers used in this work to generate *HOG1* mutant versions.

**Mutation**	**Primer**	**Sequence**
F321L	F321LHOGU	GAGCCTGTTTGTGAGAGTAAATTGGATTGGAGTTTTAATGACG
	F321LHOG1	CGTCATTAAAACTCCAATCCAATTTACTCTCACAAACAGGCTC
K52R	K52R1	CTGGTCAAAATGTTGCAGTGAGAAAAGTCATGAAACC
	K52R2	GGTTTCATGACTTTTCTCACTGCAACATTTTGACCAG
T174A-176F	TGYAGF1	CTTCAAGATCCACAAATGGCTGGTTTCGTGTCAACCAG
	TGYAGF2	CTGGTTGACACGAAACCAGCCATTTGTGGATCTTGAAC

Fluorescent labeled CAI-4 and *hog1* mutant strains were generated by integration of the pNIM1R-dTOM2 plasmid at the *ADH1 locus* (Prieto et al., 2014). This plasmid carries a Red Fluorescent Protein under the control of the tetracycline-repressible promoter. The strains obtained appear reddish on SD plates (2% glucose, 0.5% ammonium sulfate, 0.17% yeast nitrogen base) allowing quantification (visually) of their relative abundance in competition assays and differentiation from others yeast colonies.

Overexpression of cytosolic catalase in *hog1* mutants was achieve by integrating the plasmids pNRU-CAT1 or pNRU-e (empty vector) in the *ADH1* region (Román et al., 2016). pNRU-CAT1 plasmid carries the *CAT1* gene under the control of the tetracycline repressible promoter and tagged to the myc epitope. The vectors, previously digested with *Kpn*I and *Sac*II, were integrated in the *hog1* background generating the *hog1*-pNRU-CAT1 and *hog1*-pNRUe corresponding strains (Table 1).

### Killer toxin assay

*Candida albicans* overnight cultures in YPD were refreshed to an optical density of 0.8 (O.D._600nm_) in 4 mL of saline solution and spread with a sterile swab on plates of YMB medium (0.3% yeast extract, 0.3% malt extract, 0.5% peptone, 1% glucose) supplemented with 3 % NaCl and buffered with citrate phosphate to pH 4.4 or 4.8. Then, mycocins-producing strains were patched with the help of a sterile swab on the plates and incubated at 30°C (otherwise indicated) for 2 days. Alternatively, mycocins-producing strains were grown for 2 days at 30°C in liquid YPD or YMB pH 4.4; cells were then eliminated through centrifugation plus filtration through a 0.45 μm pore filter (Millipore) and 15 μL of the resulting supernatant was used to soak sterile paper filter discs (6 mm, Filter-Lab). Dried discs were then disposed on YMB 3% NaCl buffered plates previously inoculated with the *C. albicans* strain to be tested and incubated at 30°C for 2 days. Plates were photographed and the inhibition zone produced by *Dh* mycocin was measured using the free software ImageJ and expressed in millimeters (mm). Inhibition zone refers the distant between the end of *D. hansenii* growth (or disk) and the beginning of *C. albicans* growth.

### Protein extracts and immunoblot analysis

*Candida albicans* overnight cultures were refreshed to an optical density of 0.1 (O.D._600nm_) and incubated until cultures reached an optical density of 1. Cultures were then divided in three cultures and either 1 M NaCl or 10 mM hydrogen peroxide was added to the medium, the last culture remained as control. Samples were collected 10 min later and processed for Western-blotting. Protein extracts were obtained as previously indicated (Martin et al., 2000). Even amounts of proteins were loaded onto gels as assessed by protein sample estimates at 280 nm. Blots were probed with anti-phospho-p38 MAP kinase (Thr180/Tyr182) 3D7 monoclonal antibody (Cell Signaling Technology, Inc.), anti-phospho-p44/42 MAP kinase antibody (Thr202/Tyr204) (D13.14.4E) (Cell Signaling Technology, Inc.) and anti-myc Tag antibody, clone 4A6 (Millipore). The detection was performed using the Quantitative Fluorescent Imaging System Odyssey from Li-COR.

### Flow cytometry

Flow cytometry was used to quantify intracellular Reactive Oxygen Species (ROS) using dihydrofluorescein diacetate (DHF) and mitochondrial membrane potential using rhodamine 123 (R123). Briefly, overnight cultures from the different *C. albicans* strains were collected by centrifugation and refreshed either in pre warmed YMB pH 4.4 medium at 30°C or in *Dh*-242 overnight spent medium. *C. albicans* cultures were incubated at 30°C for 1 and 2 h. Thirty minutes before this time DHF or R123 was added to the samples to a final concentration of 40 and 20 μM respectively and incubated at 30°C in the dark for the remaining 30 min. Samples were collected, washed twice with PBS and resuspended in PBS at 10^6^ cells per mL. Then, propidium iodide (IP) was added to detect dead cells. Fluorescence intensity was determined by flow cytometry using the FACScan cytometer (Beckton Dickinson) from the Servicio de Citometría (Universidad Complutense de Madrid). The analysis of green (FL1) fluorescence intensity was done on a logarithmic scale and gates were set around debris and intact cells on a FSC vs. SSC dot plot. Positive IP cells (dead cells) were removed from the analysis. Fluorescence histograms corresponding to 10,000 cells were generated using the gated data with data analyses done using Flowing Software 2.5.1.

### Glycerol quantification

To quantify the intracellular glycerol, exponentially growing cells were centrifuged and resuspended in YMB 3% NaCl pH4.4 medium supplemented with 1 M NaCl (positive control) or the medium from *Dh*-242 strain grown at 30°C during 48 h. Samples were taken before and 1 and 3 h after the challenge. Ten milliliter of the culture was harvested and filtered through a 0.45 μm filter previously weighed. The dry weight was determined by subtracting the weight of the filter from the weight obtained after drying the filtered pellets for 2 days at 45°C until stable weight. For glycerol determination, a 1 mL sample was centrifuged at 10,000 rpm for 5 min; the pellet was resuspended in 1 mL of ultrapure water (Milli Q) and boiled at 99°C for 10 min. Cell debris were removed by centrifugation (10 min 10,000 rpm) and the supernatant was collected. The amount of glycerol was determined using the commercial preparation of Megazyme Glycerol Assay Kit (K-GCEROL following manufacturer instructions. Intracellular glycerol is expressed in μg of glycerol per mg of dry weight.

## Results

### *C. albicans hog1* mutants are sensitive to killer toxins from different *D. hansenii* strains

*Debaryomyces hansenii* strains previously reported to have anti-candida activity (Banjara et al., 2016) were tested against the *C. albicans* SC5314 strain at different pHs and temperatures (Table 4 and Supplementary Figure 1). Although no killer activity was found at 37 or 37°C plus hypoxia, a clear killer effect (as determined by the halo) was observed when different *D. hansenii* strains were tested at 24 and 30°C, being higher at pH 4.4 than at pH 4.8 (Table 4).

**Table 4:** Killer effect *of D. hansenii* strains against *C. albicans* strains.

		**Dh-65**	**Dh-72**	**Dh-220**	**Dh-242**	**Dh-246**	**Dh-262**	**Dh-274**	**CBS-767**
SC5314 (wt)	pH 4.4	1.79 ± 0.25	1.31 ± 0.09	–	1.92 ± 0.24	1.07 ± 0.19	0.97 ± 0.09	0.72 ± 0.11	0.93 ± 0.20
	pH 4.8	1.41 ± 0.12	–	–	0.77 ± 0.22	0.95 ± 0.06	0.64 ± 0.15	–	0.74 ± 0.13
*mkc1*	pH 4.4	1.02 ± 0.2	–	–	1.15 ± 0.34	1.50 ± 0.70	1.18 ± 0.20	1.39 ± 0.11	1.06 ± 0.32
	pH 4.8	0.29 ± 0.05	–	–	–	0.69 ± 0.08	0.84 ± 0.06	0.67 ± 0.21	0.93 ± 0, 21
*hog1*	pH 4.4	4.82 ± 0.15	0.88 ± 0.03	2.42 ± 021	4.70 ± 0.30	6.72 ± 0.48	6.48 ± 0.06	6.23 ± 0.60	5.36 ± 0.42
	pH 4.8	4.32 ± 0.25	2,.3 ± 0.34	2.49 ± 0.43	3.37 ± 0.28	3.6 ± 0.30	3.37 ± 0.21	3.70 ± 0.12	3.43 ± 0.04
*cek1*	pH 4.4	1.17 ± 0.25	–	–	1.12 ± 0.18	1.08 ± 0.71	1.37 ± 0.67	0.86 ± 0.1	1.10 ± 0.18
	pH 4.8	–	–	–	1.27 ± 0.11	0.76 ± 0.08	0.80 ± 0.10	0.73 ± 0.06	1.15 ± 0.18

*Inhibition zone was measured in mm. The assay was performed on YMB 3% NaCl at pH 4.4 or 4.8 plates and incubated at 30°C. Data are the mean of two independent experiments ± Standard deviation. Three measures were performed per experiments*.

**Figure 1:**
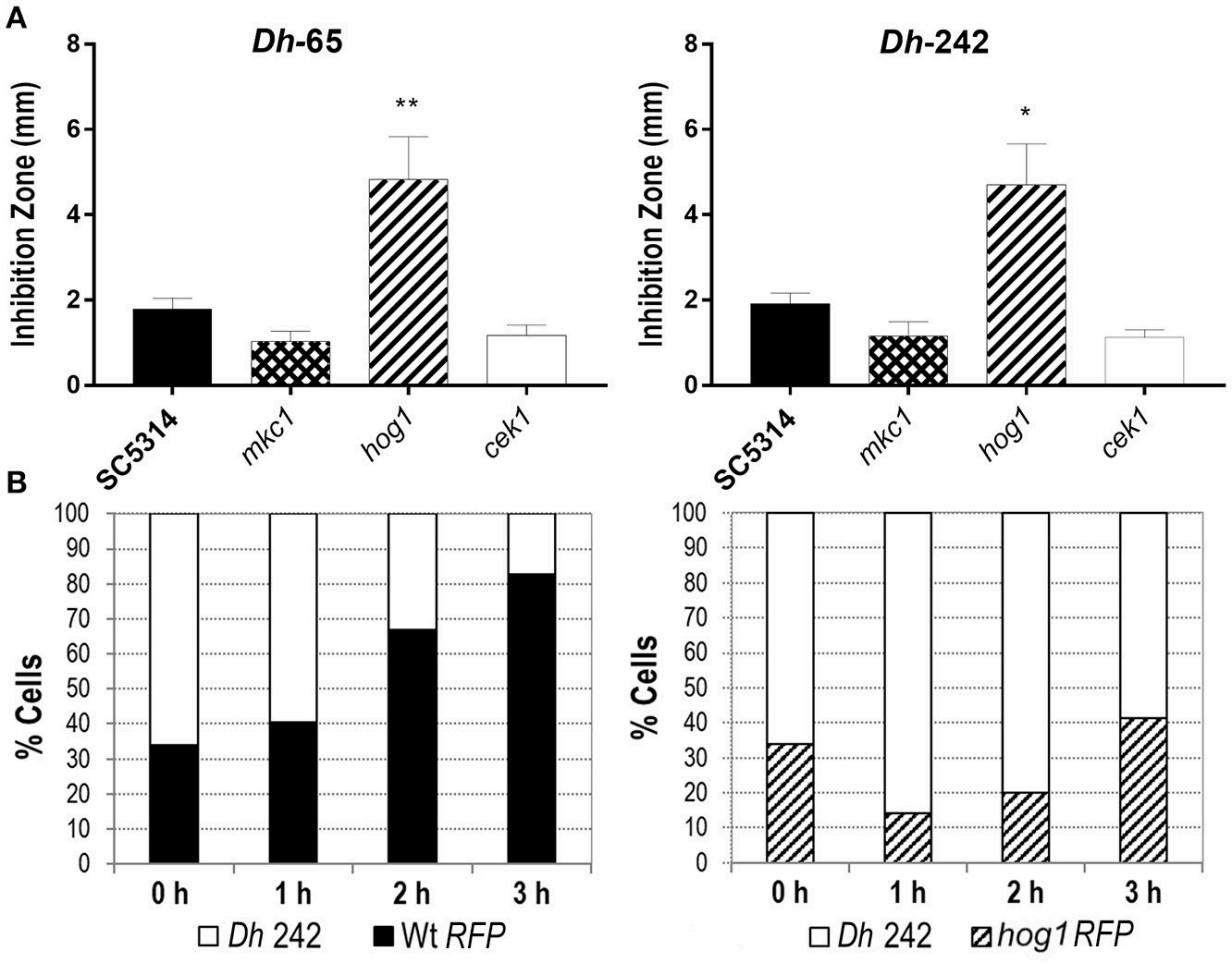
Effect of *D. hansenii* (Dh) strains on *C. albicans* growth. **(A)** The killer activity of *Dh*-65 and *Dh*-242 strains was tested against the indicated *C. albicans* strains on YMB 3% NaCl pH 4.4 plates at 30°C for 48 h. Graph shows the mean and *SD* from three independent experiments. *t*-test analyses were performed to show statistical significant differences. ^**^p < 0.01 and ^*^p < 0.05. **(B)** *C. albicans* strains carrying the fluorescent protein dTOM2 (RFP) were mixed with *Dh*-242 strain to 1:2 proportion in YPD pH 4.4 and incubated at 30°C with shaking. Samples were collected at indicated time points and CFUs were counted and expressed as percentages. RFP label allows differentiating *C. albicans* (red colonies) from *D. hansenii* (white colonies).

In order to characterize the mechanism of action and the targets of these *Dh* killer toxins we examined the behavior of *C. albicans* MAPKs defective mutants in the presence of the mycocin-producing *D. hansenii* strains. The mutant lacking the Hog1 MAPK displayed an enhanced susceptibility to most of *D. hansenii* strains tested while this was not observed for mutants defective in either Cek1 or Mkc1 (Table 4 and Figure 1A). In fact, *cek1* and *mkc1* mutants were slightly more resistant than the control strain to killer toxins from *Dh*-65, *Dh*-72, *Dh*-220, and –partially- to *Dh*-242 although these effects were not statistically significant (Table 4 and Figure 1A).

*Dh*-242 strain was chosen to analyze its effect on *C. albicans* liquid cultures since the killing effect of this strain was clear against the strain analyzed. Wild type and *hog1 C. albicans* strains were tagged with the fluorescent protein dTOM2 in order to discriminate between *C. albicans* and *D. hansenii* cells. *C. albicans* strains tagged with dTOM2 (Prieto et al., 2014) were mixed to a 1:2 proportion with the strain *Dh*-242 and incubated together at 30°C. Samples were collected after 1, 2 and 3 h, dilutions spread on *SD* plates and white and red colonies counted and expressed as percentage (Figure 1B). The *C. albicans* wild type strain was able to grow in the presence of mycocin-producer *Dh* strain from the first hour, being more than 80 % of the culture after 3 h of incubation. In the case of *hog1* cultures, the proportion of *hog1* cells decreased to 14% after 1 h and then, resumed growth increasing the percentage of *C. albicans* cells to the detriment of *Dh* strain reaching 40% of the total cells in culture. This result indicates that *Dh*-242 strain has an inhibitory effect on *C. albicans hog1* mutant in liquid medium that did not impair the wild type strain. *Dh*-242 compromised *hog1* mutant survival at short time after co-incubation (1–2 h). Thus, the *Dh*-242 effect is enhanced in the *hog1* mutant suggesting that the HOG pathway could be involved in the response to mycocins produced by this strain.

### *Dh*-242 supernatant induces glycerol accumulation but not oxidative stress in *C. albicans*

**Figure 2:**
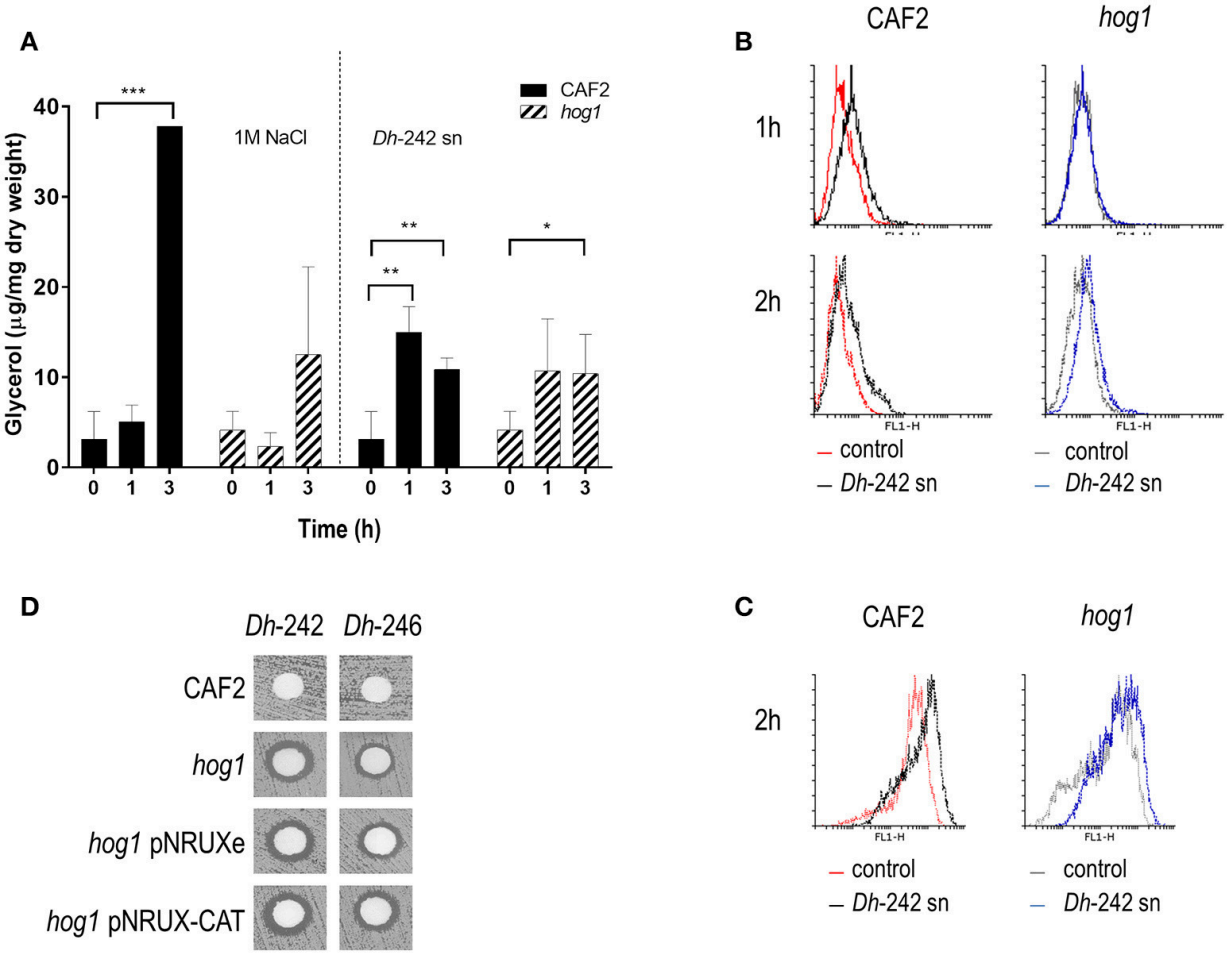
Effect of *Dh*-242 supernatant on intracellular glycerol accumulation and oxidative stress. **(A)** *C. albicans* wild type and *hog1* mutant cells were resuspended in either a supernatant from *Dh*-242 strain grown for 48 h in YMB 3% NaCl pH 4.4 or fresh medium pre-warmed at 30°C supplemented with 1M NaCl (1.5 M final concentration). Samples were collected at 0, 1, and 3 h and intracellular glycerol was quantified. Graph shows the mean and SD from 3 independent experiments. *t*-test analyses were performed to show statistical significant differences ^***^p < 0.001, ^**^p < 0.01, and ^*^p < 0.05 **(B)** CAF2 and *hog1* mutants strains were incubated in the presence of supernatant from *Dh*-242 strain (+) or YMB pH 4.4 (−) liquid medium at 30°C. Intracellular oxidative stress was quantified by flow cytometry using Rhodamine 123. A histogram from a representative experiment is shown. **(C)** Mitochondrial membrane potential was quantified using DHF after 2 h of incubation in supernatant from *Dh*-242 strain (+) or YMB pH 4.4 (−) liquid medium. A histogram from a representative experiment is shown. **(D)** A killer toxin assay was performed using supernatant from *Dh*-242 and *Dh*-246 strains grown in YMB 3% NaCl pH4.4 liquid medium for 48 h. The *hog1* mutant strain carrying the empty vector (pNRUXe) or overexpressing the Catalase (pNRUX-CAT) were tested.

In order to check if *Dh*-242 KT altered cell permeability promoting an osmotic stress that triggers the HOG pathway as it has been suggested for other killer toxins (Santos and Marquina, 2011), we measured the intracellular accumulation of glycerol in response to *Dh*-242 supernatant. A wild type strain increased the intracellular glycerol in the presence of 1 M NaCl reaching a maximum after 3 h of incubation. I In the presence of *Dh*-242 supernatant the accumulation was earlier (maximum at 1 h) and transient since it decreased after 3 h of incubation (Figure 2A). The *hog1* mutant accumulated intracellular glycerol in the presence of *Dh* supernatant to a similar level as the wild type strain (Figure 2A). No glycerol accumulation was detected when cells were grown in YMB 3% NaCl pH 4.4 indicating that 3% NaCl (0.5M) is not enough to induce glycerol accumulation in *C. albicans* and that the observed effect is due to the *Dh* killer toxin (Supplementary Figure 2). These results suggest that *Dh* mycocins present in the supernatant of *Dh*-242 caused an osmotic stress in *C. albicans* cells triggering a transient accumulation of glycerol which is independent of Hog1.

Since the HOG pathway is also needed to respond to oxidative stress, the intracellular ROS amount was measured using flow cytometry analysis in response to a supernatant from the *Dh*-242 strain. Wild type and *hog1* strains showed a similar percentage of cells stained with IP (dead, IP+) within the first 2 h. In the presence of *Dh* supernatant, cultures of the CAF2 wild type strain increased intracellular ROS at 1 and 2 h while in the case of the *hog1* mutant was less evident even after 2 h of incubation (Figure 2B). Since intracellular ROS accumulation requires mitochondrial activity the mitochondrial membrane potential was also quantified by flow cytometry. The mitochondrial membrane potential in the presence of the *Dh*-242 supernatant increased equally in both strains after 2 h in the presence of the *Dh*-242 supernatant when measured with DHF (dihydrofluorescein) (Figure 2C).

Since the differences in intracellular ROS and mitochondrial membrane potential were not substantial, we checked if a *hog1* mutant overexpressing catalase (*CAT1*) could overcome the effect of *Dh*-KT (Figure 2D). *C. albicans* cells overexpressing *CAT1* are more resistant to hydrogen peroxide, phagocytes, and decrease intracellular ROS generated by antifungal such as amphotericin B (Román et al., 2016). The *hog1* mutant strain was transformed with the empty vector or with a vector carrying the *CAT1* gene under the control of tetracycline promoter in its repressible version. The overexpression of *CAT1* did not revert the sensibility to *Dh*-KT displayed by the *hog1* mutant.

Altogether, we can conclude that the increased sensitivity of the *hog1* mutant to *Dh* mycocins is not due to the impairment of the response to osmotic or oxidative stress mediated by *HOG1*, pointing to other type of defects.

### The MAPK Hog1 but not the HOG pathway is essential for killer toxin activity

As certain MAPK mutants displayed altered susceptibilities to killer toxins, mutants in other elements of the HOG, cell wall integrity (*MKC1*) and Cek1-mediated pathways were analyzed under similar conditions (Table 5 and Supplementary Figure 3). Remarkably, the deletion of the response regulator of two-component system (Ssk1), the MAPKK (Pbs2), or the transcription factor (Sko1) which participate in the HOG pathway displayed variable susceptibility to *Dh* mycocins compared to the parental wild type strain (RM100) (Table 5). Interestingly, none of the mutants showed sensitivities to *Dh*-mycocins to the same extend that the *hog1* mutant in the same background (CNC13 strain). The CNC13 strain displayed an enhanced sensitivity compared to the parental strain RM100, suggesting that the sensitivity to *Dh* killer toxins does not depend on the genetic background but, rather, it is specific to *HOG1* deletion. As expected, the integration of the *HOG1* gene into the *hog1* mutant restored wild type phenotype. When *pbs2 hog1* and *sko1 hog1* double mutants were tested, the susceptibility to mycocins depended on the *Dh* strain analyzed (Table 5). *pbs2 hog1* and *sko1 hog1* double mutants were more susceptible than the *hog1* mutant to *Dh*-220 and *Dh*-274 mycocins. The inhibition zone in the presence of the control strain CSB-767 was reduced in *pbs2 hog1* and *sko1 hog1* double mutants compared to *hog1* strain. These data suggest that Hog1 is crucial for the tolerance to *D. hansenii* killer toxins, while, others elements of the pathways such as Ssk1, Pbs2 and Sko1 play a relatively minor role in killer toxin tolerance. Mutants in upstream elements of the Cek1 pathway such as the transmembrane proteins Msb2, Opy2 or the MAPKK of the Mkc1 pathway, Mkk2 were more resistant to certain *Dh* killer toxins than the parental strain (Table 5) suggesting that these mutants lack (or have in lesser proportion) a putative receptor(s) required to bind or activate *Dh* killer toxins.

**Table 5:** Killer effect *of D. hansenii* strains against *C. albicans* strains.

	***Dh*-65**	***Dh*-72**	***Dh*-220**	***Dh*-242**	***Dh*-246**	***Dh*-262**	***Dh*-274**	**CBS-767**
RM100	0.68 ± 0.23	–	0.75 ± 0.06	0.83 ± 0.21	0.95 ± 0.14	1.01±.16	0.73 ± 0.09	0.95 ± 0.1
*ssk1*	0.65 ± 0.14	–	–	0.87 ± 0.17	1.32 ± 0.09	0.67 ± 0.08	1.24 ± 0.19	0.67 ± 0.07
*pbs2*	0.67 ± 0.07	–	–	1.84 ± 0.10	1.86 ± 0.05	1.83 ± 0.27	2.99 ± 0.23	0.51 ± 0, 26
*hog1* (RM100)	6.78 ± 0.01	2.71 ± 0.19	2.97 ± 0.49	4.34 ± 0.28	6.37 ± 0.16	6.36 ± 0.38	5.12 ± 0.59	6.00 ± 0.16
*hog1^*reint*^* (CNC16-13)	1.54 ± 0.32	–	–	1.77 ± 0.17	1.09 ± 0.13	1.02 ± 0.17	0.93 ± 0.16	0.63 ± 0.07
*hog1 pbs2*	4.26 ± 0.14	3.60 ± 0.26	6.25 ± 0.26	4.67 ± 0.03	6.20 ± 0.46	6.87 ± 0.38	6.51 ± 0.45	5.31 ± 0.25
*sko1*	0.86 ± 0.33	–	0.57 ± 0.38	1.45 ± 0.59	1.49 ± 0.69	1.63 ± 0.69	1.26 ± 0.61	0.83 ± 0.185
*hog1 sko1*	2.02 ± 0.24	3.31 ± 0.62	8.1 ± 0.23	1.82 ± 0.09	5.93 ± 0.39	6.98 ± 0.35	5.59 ± 0.32	1.59 ± 0.24
*msb2*	–	–	–	0.37 ± 0.01	0.57 ± 0.11	0.5 ± 0.14	0.42 ± 0.03	0.44 ± 0.07
*opy2*	–	–	–	1.63 ± 0.40	1.19 ± 0.11	1.856 ± 0.12	0.82 ± 0.05	–
*mkk2*	–	–	–	0.60 ± 0.06	0.50 ± 0.19	0.58 ± 0.19	–	–

*Inhibition zone was measured in mm. The assay was performed on YMB 3% NaCl at pH 4.4 plates and incubated at 30°C. Data are the mean of two independent experiments ± Standard deviation. Three measures were performed per experiments*.

### Hog1 phosphorylation and kinase activity are crucial for killer activity resistance

**Figure 3:**
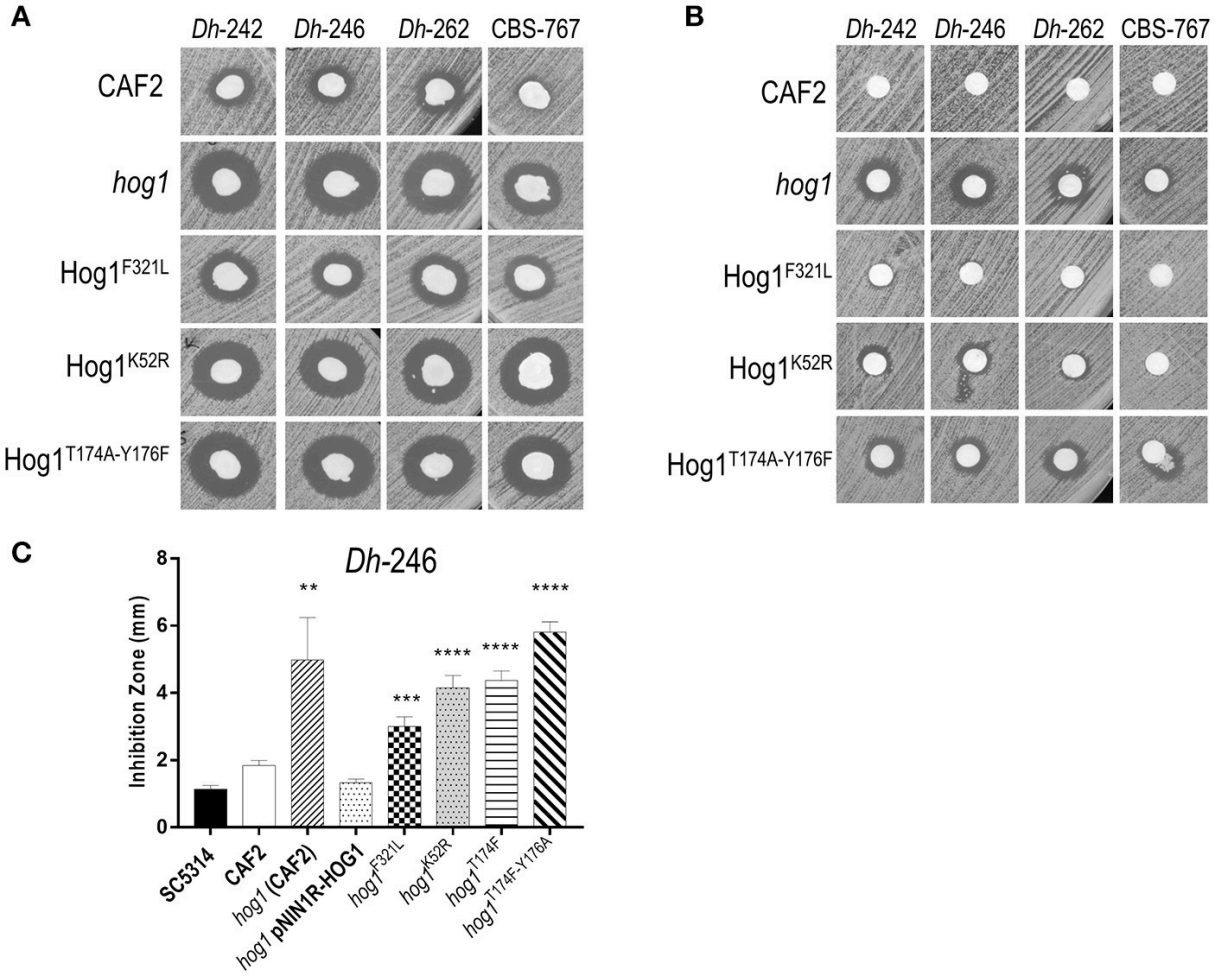
Effect of *D. hansenii* killer strains on different *hog1* mutants. **(A)** Killer toxin assay of different *D. hansenii* strains (*Dh*) against different *C. albicans* strains. Equal suspensions of *C. albicans* strains were spread on YMB plates supplemented with 3% NaCl pH 4.4 with a sterile swab and, *D. hansenii* strains were immediately spotted with a swab; the plates were thenincubated at 30°C **(B)** Killer toxin assay of supernatants of different *D. hansenii* strains against the indicated *C. albicans* strains. *Dh* strains were grown in YMB 3% NaCl pH 4.4 liquid medium for 48 h, centrifuged and 15 μl of the filtered supernatant were inoculated in a paper filters. Paper filters impregnated with the supernatant were deposited on YMB 3% NaCl pH 4.4 plates previously inoculated with the indicated *C. albicans* strains using a sterile swab. **(C)** Killer activity of the *Dh*-246 strain against different *C. albicans* strains was measured (in mm) from at least three independent experiments. *t*-test analyses were performed to show statistical significant differences. ^****^p < 0.001, ^***^p < 0.001, and ^**^p < 0.05.

Since upstream and downstream elements of the HOG pathway played a limited role in sensitivity to *Dh*-242 supernatant, we checked if the regulation module or the kinase activity of Hog1p could be important for this effect by obtaining different mutants and testing their killer toxin susceptibility. The lysine in position 52 was replaced by an arginine to generate a mutant defective in its kinase activity (Hog1^K52R^). Similarly, the phosphorylation site, threonine 174 and tyrosine 176, were replaced by alanine and phenylalanine, respectively, to generate a mutant unable to be phosphorylated in its regulation domain (Hog1^T174A Y176F^). In addition, a single substitution of one of those phosphorylation sites was also obtained (Hog1^T174A^). Finally, a phosphomimetic Hog1 version was generated replacing the phenylalanine 321 by a leucine (Hog1^F321L^). Both kinase dead (Hog1^K52R^) and non-phosphorylatable (Hog1^T174A Y176F^) strains were found to be susceptible to killer toxins in a similar magnitude to the *hog1* mutant (Figure 3A). Moreover, phosphomimetic Hog1 version (Hog1^F321L^) displayed an intermediate susceptibility between the *hog1* mutant and wild type strain, suggesting that dephosphorylation of Hog1 must be relevant to Hog1 function (Figure 3A). Also, *HOG1* overexpression reverted the sensitivity to mycocins to wild type levels, while the Hog1^T174A^ strain behaved similarly to the kinase dead mutant (Figure 3C). Similar results were obtained in the presence of other mycocin-producing *D. hansenii* strains (Data not shown).

In parallel, we tested the killer activity exerted by supernatants from *Dh* mycocin-producing strains to confirm that *Dh* mycocin were secreted to the culture medium (Figure 3B). Although no killer effect was detected against wild type *C. albicans, hog1*, and Hog1^T174A Y176F^ strains displayed a clear inhibition zone (Figure 3B). The kinase dead strain (Hog1^K52R^) showed a weak inhibition halo, suggesting that Hog1 phosphorylation is more important than kinase activity to tolerate *Dh* mycocins.

### Mutations in *HOG1* induce an altered congo red sensitivity

**Figure 4:**
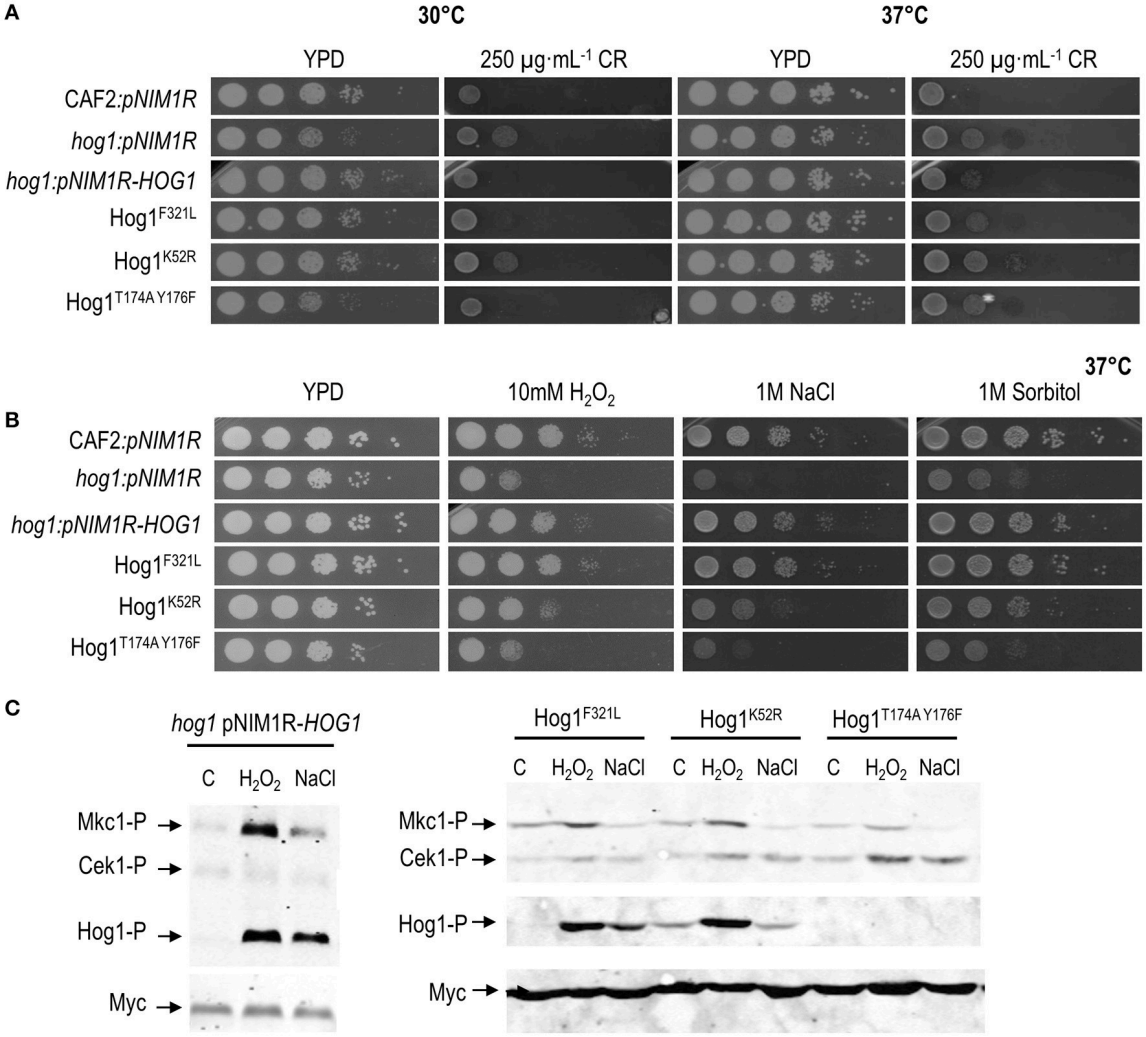
Role of different domains of Hog1 in tolerance and response to stress. **(A)** *C. albicans* strains carrying different *HOG1* gene versions were spotted on YPD plates supplemented or not with Congo red and incubated for 24 h at 30 or 37°C. **(B)** Tenfold cell suspensions of the indicated strains were spotted on YPD solid medium supplemented with the indicated compounds and incubated at 37°C for 24 h. **(C)** Exponentially growing cells were exposed to 10 mM H_2_O_2_ or 1M NaCl during 10 min and processed for immunoblot detection. Mkc1-P, Cek1-P, and Hog1-P indicate the phosphorylated forms of the MAPKs. Load control was detected with Anti-Myc antibody.

Mycocins can bind different components of the fungal cell wall as first step to get the final target: β-1,3, β-1,6-D-glucan, chitin or mannans have been reported as primary receptors (Liu et al., 2015). Since alterations in cell wall biogenesis have been described in *hog1* mutants (Alonso-Monge et al., 1999), we wondered if there was any correlation between sensitivity to *Dh* killer toxins and others cell wall-related phenotypes. Susceptibility to caspofungin and Congo red was tested on plates at 30 and 37°C. *hog1* mutants did not display caspofungin sensitivity compared to wild type at any temperature and condition analyzed (Supplementary Figure 4). Nevertheless, *hog1* and Hog1^K52R^ strains are resistant to Congo red at both temperatures (30 and 37°C) (Figure 4A). The non-phosphorylatable Hog1^T174A^ ^Y176F^ strain displayed Congo red resistance at 37°C and sensitivity at 30°C (the temperature used to perform killer toxin activity). Therefore, the lack of Hog1 renders cells sensitive to *Dh* killer toxins and resistant to Congo red and this phenotype depends on its kinase activity.

The susceptibility to cell wall disturbing compounds was also analyzed for others signaling mutants (*mkc1, mkk2, cek1*, and *opy2*) resistant to *Dh*-mycocins. In agreement with the correlation between resistance to killer toxin and Congo red sensitivity, these mutants are more sensitive to Congo red than the wild type strain (Herrero de Dios et al., 2013; Román et al., 2015) (Supplementary Figure 5).

Since the kinase activity of Hog1 seems to be important for Congo red resistance, we tested the role played by specific Hog1 domains in the canonical Hog1 functions, signaling and adaptation to osmotic and oxidative stress. Sensitivity assays were performed by spotting cell suspensions on YPD plates supplemented with H_2_O_2_, NaCl or sorbitol, and incubated at 37°C. As shown in Figure 4B, the non-phosphorylatable Hog1^T174A Y176F^ strain displayed an increased susceptibility to oxidative and osmotic stress similar to the *hog1* deletion mutant. Oddly, the kinase-dead strain expressing Hog1^K52R^ displayed an intermediate phenotype between wild type and the *hog1* deletion mutant. Mutants carrying the phosphomimetic Hog1 version or overexpressing *HOG1* behaved as the wild type strain and did not increase the tolerance to osmotic and oxidative stress.

The MAPK phosphorylation pattern was also analyzed in response to standard stimuli that trigger the HOG pathway: hydrogen peroxide and NaCl (Figure 4C). In response to oxidative stress, Hog1 was phosphorylated in phosphomimetic and the kinase-dead Hog1 strains. In response to osmotic stress, Hog1 phosphorylation was clear in Hog1^F321L^ strain although the kinase dead (Hog1^K52R^) strain showed a lower phosphorylation level in response to NaCl. As expected, no Hog1 phosphorylation was detected in non-phosphorylatable Hog1^174A Y176F^ strain. Moreover, Mkc1 was phosphorylated in response to hydrogen peroxide in both the phosphomimetic and the kinase-dead Hog1 strains. The non-phosphorylatable Hog1 strain displayed higher Cek1 phosphorylated level and hardly activated Mkc1 in response to H_2_O_2_. This MAPK phosphorylation pattern matches with *hog1* deletion mutant (Arana et al., 2005; Alonso-Monge et al., 2009). These data indicate that Hog1 phosphorylation is required to trigger osmotic and oxidative stress responses that enable *C. albicans* to grow in the presence of these stresses. The *HOG1* overexpressing strain (*hog1* pNIM1R-HOG1 strain) behaved as the wild type strain (Figure 4B left panel).

## Discussion

*Candida albicans* infections represent a major health problem worldwide. In spite of the introduction of extended-spectrum triazole and echinocandin antifungal agents in the last decades, the frequency of candidemia and others invasive candidiasis, as well as the associated mortality (reaching 49% of patients) have not decreased. Current research efforts focus on finding faster and more sensitive diagnostic methods to avoid unnecessary treatments, fighting against antifungal resistance and reducing economic costs (Pfaller and Castanheira, 2016). Searching for novel antimycotics or new natural compounds with anti-*Candida* activity is an interesting alternative approach. For example, several phytocompounds have been proved to exert anti-*Candida* activity, some of them with synergistic effect in combination with fluconazole (Lu et al., 2017). In addition, different killer toxins have been discovered to have activity against *C. albicans* (Liu et al., 2015).

We focused here on some *D. hansenii* strains isolated from cheese displaying activity against *Candida* (Banjara et al., 2016), although the molecular basis for this activity have not been reported. Our results indicate that these killer toxins present an optimum inhibitory effect at pH 4.4, 30°C and in NaCl-supplemented medium, similarly to what occurs with most of the killer toxins reported (with few exceptions) (Liu et al., 2015).

We found that the absence of the Hog1 MAPKK promoted sensitivity to mycocins from several *D. hansenii* strains. Unexpectedly, this was only detected in the *hog1* mutant and not in others mutants of the pathway such as *pbs2, ssk1* or *sko1*. Hog1 phosphorylation depends directly on the MAPKK Pbs2 (Arana et al., 2005) and consequently, *hog1* and *pbs2* mutants share sensitivity to those stresses that trigger Hog1 phosphorylation such as osmotic and oxidative stress. This suggests that the susceptibility to *Dh* killer toxins seems to be more complex and may involve different triggering stimuli. What differentiates *hog1* from *pb*s2 mutants? One possibility is the cell wall architecture: *hog1* and *pbs2* mutants share several phenotypes but not sensitivity to cell wall disturbing compounds. The lack of Hog1 renders the mutant more resistant to calcofluor white and Congo red than the wild type, *pbs2* mutant (Arana et al., 2005) and the *sko1* mutant (Alonso-Monge et al., 2010). Killer toxins require two receptors to exert their effect: the primary receptor is usually located on the cell wall and the secondary receptor on the plasma membrane or cytosol. The first step for killing activity involves the binding of the mycocin to a yet unknown cell wall component. This component can be any of the constituents that integrate the fungal cell wall such as β-1,3 or β-1,6-D-glucan, chitin or mannans (Liu et al., 2015). Our data indicate that there is no relationship between caspofungin and *Dh* killer toxin sensitivity but there is with Congo red resistance. Since no difference could be found in caspofungin susceptibility between Hog1 mutants and wild type strains, the compensatory mechanism of cell wall construction that increase chitin accumulation should not be responsible for the different sensitivity to *Dh* killer toxin (Lee et al., 2012). *C. albicans hog1* mutants have been shown to be more sensitive to zymolyase (Eisman et al., 2006), a β-1,3-glucanase preparation with a residual protease activity that is essential to allow the access of the β-1,3-glucanase to its target (Zlotnik et al., 1984; García et al., 2009). *hog1* mutants have an increased β-1,3-glucan exposure (D. Prieto unpublished data), suggesting an altered cell wall that could in turn be responsible for increased access of zymolyase (and maybe also mycocins) to its target, probably β-1,3-glucan.

After reaching the cytoplasmic membrane certain killer toxins, such as the K1 toxin produced by *S. cerevisiae*, exert its lethal activity by ion channel formation and therefore, perturbing the permeability of the membrane (Flegelová et al., 2003). Similarly, the killer toxin from *P. membranifaciens* PMKT has been suggested to bind to a receptor in the cell wall such as β-D-(1,6)-glucan that would somehow allow crossing the cell wall and binding to a second receptor in the plasma membrane (Santos et al., 2005; Santos and Marquina, 2011). PMKT causes the loss of ions and metabolites which is sensed by yeast cells as a hyperosmotic stress therefore triggering Hog1p phosphorylation (Santos and Marquina, 2011). Interestingly, the supernatant from *Dh*-242 strain also induces a transient intracellular glycerol increase in *C. albicans*, suggesting that *Dh*-242 mycocin may trigger an osmotic stress response which would be Hog1-independent. In agreement with this, no phosphorylation of Hog1 was detected when *C. albicans* was exposed to *Dh* supernatants (data not shown). This could be caused by the reduced mycocin concentrations in the supernatant, not enough to trigger the response. However, it can be also possible that *Dh* mycocins do not trigger Hog1 phosphorylation in *C. albicans*. Glycerol accumulation could be due to the closure of channels which would in turn prevent glycerol efflux through a mechanism independent of Hog1 as occurs in *S. cerevisiae* (Saxena and Sitaraman, 2016). Although these results lead us to conclude that the participation of Hog1 in the response to the osmotic stress generated by *Dh*-242 mycocin is not crucial, the assays performed on solid medium indicate that Hog1 phosphorylation is needed. The continued presence of *Dh* cells and therefore, *Dh* mycocins, may generate a sustained osmotic stress that requires Hog1 phosphorylation.

The relevance of the phosphorylation domain for Hog1 activity was reported previously (Cheetham et al., 2011; Chang et al., 2016) but these previous works did not address the role of the kinase domain as shown here. Interestingly, the Hog1 kinase dead mutant behaves closer to the wild type strain under osmotic and oxidative stress but closer to the *hog1* deletion mutant on Congo red supplemented plates. Thus, cell wall related phenotypes require Hog1 kinase activity although Hog1 phosphorylation is not so relevant. This suggests that non-phosphorylated (or basal Hog1 phosphorylation) is enough to control cell wall biogenesis, maybe controlling others MAPK phosphorylation (in the absence of Hog1, Cek1 is constitutively phosphorylated) or its own pathway as reported in *S. cerevisiae* (Sharifian et al., 2015). Survival of *C. albicans* cells when exposed to *Dh*-killer toxins requires both phosphorylation and kinase activities indicating that both the role of Hog1 in cell wall biogenesis and signaling are compulsory to face *Dh*-mycocins.

The phosphorylation of the kinase dead Hog1 mutant may, somehow, be enough to trigger a downstream response and bypass the requirement for kinase activity. This observation is in agreement with results reported previously that showed that the nuclear localization of Hog1 is dispensable for many of the functions exerted by this MAPK (Day et al., 2017) such as growth in the presence of osmotic or oxidative stressors, virulence or gene transcription. Hog1 localization is, however, important to maintain signal fidelity, avoiding others MAPKs being phosphorylated that could change cell wall architecture. How Hog1 can trigger a downstream cascade without being translocated to the nucleus or without kinase activity remains unknown. The phosphomimetic version of Hog1 (Hog1^F321L^) did not increase the resistance to different stresses nor triggered Hog1 constitutive phosphorylation but became phosphorylated when exposed to osmotic or oxidative stress. A similar behavior was described by Cheetham and co-workers analyzing a phosphomimetic version of the MAPKK Pbs2 (Pbs2^DD^) (Cheetham et al., 2011); a Pbs2^DD^ allele did not phosphorylate Hog1 constitutively, exhibited a partial sensitivity to stress and triggered Hog1 phosphorylation upon different stresses to significantly lower levels compared to wild type and this phosphorylation was independent of Ssk2 (Cheetham et al., 2011). Hyperactivation of Hog1 leads to a lethal phenotype in *S. cerevisiae* (Wurgler-Murphy et al., 1997; Yaakov et al., 2003), but not in *C. albicans*, suggesting that *C. albicans* may have evolved to prevent Hog1 constitutive activation and evidencing substantial differences in signal transduction between both yeast.

Since relationships among microorganisms in natural environments are complex and involve synergistic and antagonistic interactions, the production of killer toxins could provide evolutionary advantages for mycocin-producing strains like *D. hansenii*, a safe microorganism present in cheeses and fermented sausages as part of their production processes. *D. hansenii* strains have been isolated from the human gastrointestinal tract following ingestion of dairy products (Cosentino et al., 2001; Vasdinyei and Deák, 2003; Borelli et al., 2006; Desnos-Ollivier et al., 2008; Hallen-Adams et al., 2015). The interaction of *D. hansenii* and *C. albicans* could take place in the human gastrointestinal tract through feeding, although killer activity in the gastrointestinal tract has not been yet proved. The MAPK Hog1 seems to mediate the tolerance to killer toxins first avoiding the binding of killer toxins to cell surface and then, triggering a response to counterbalance their effects. All these data reinforce the relevance of signaling pathways in intra-species relationships and cell survival as well as the search for killer toxins that could be active at physiological temperatures in the human body.

## Author contributions

AM-M, IC, JG-A, FN-G performed the experiments. FN-G, ER, DP JP, RA-M analyzed the data, results discussion and supervising the manuscript. JP did funding acquisition. RA-M did experimental design, supervising, and writing manuscript.

### Conflict of interest statement

The authors declare that the research was conducted in the absence of any commercial or financial relationships that could be construed as a potential conflict of interest.

## Supplementary material

Supplementary Figure 1 *Dh* Kiler Toxin activity against *C. albicans* strains: The killer activity was analyzed on YMB plates supplemented with 3% NaCl pH 4.4 against SC5314 and the *hog1* mutant strain under different environmental conditions.

Supplementary Figure 2 Quantification of intracellular glycerol from CAF2 and *hog1* mutant exposed to YMB plates plus 3% NaCl pH 4.4 (as control), supplemented with 1 M NaCl or *Dh*-242 supernatant at 0, 1 and 3 h. Fold increase is shown compared to glycerol level at time 0 for each strain.

Supplementary Figure 3 Schematic graph of *C. albicans* MAPK signal transduction pathways analyzed in the present work. Transmembrane proteins and sensors are shown as rectangles, intermediate molecules are depicted as circles and MAP Kinase modules are ovals. The transcription factor Sko1 is shown as a white rectangle. Numbers indicate the inhibition zone (in mm) displayed by defective mutants in the specific elements in the presence of *Dh*-242 strain in YMB NaCl pH 4.4 at 30°C.

Supplementary Figure 4 Susceptibility of different strains to caspofungin. The indicated strains were spotted on YPD supplemented or not with caspofungin (Casp) and incubated at 30 or 37°C for 24 h before scanning.

Supplementary Figure 5 The indicated strains were spotted on YPD supplemented or not with Congo red and incubated at 30 or 37°C for 24 h before scanning.

## Abbreviations

MAPK: Mitogen Activated Protein Kinase.
