# Supplementary figures and images for: Non-canonical Activities of Hog1 Control Sensitivity of Candida albicans to Killer Toxins From Debaryomyces hansenii
Source: Front Cell Infect Microbiol. 2018 May 3;8:135. doi: 10.3389/fcimb.2018.00135 (PMC5943613; doi:10.3389/fcimb.2018.00135)

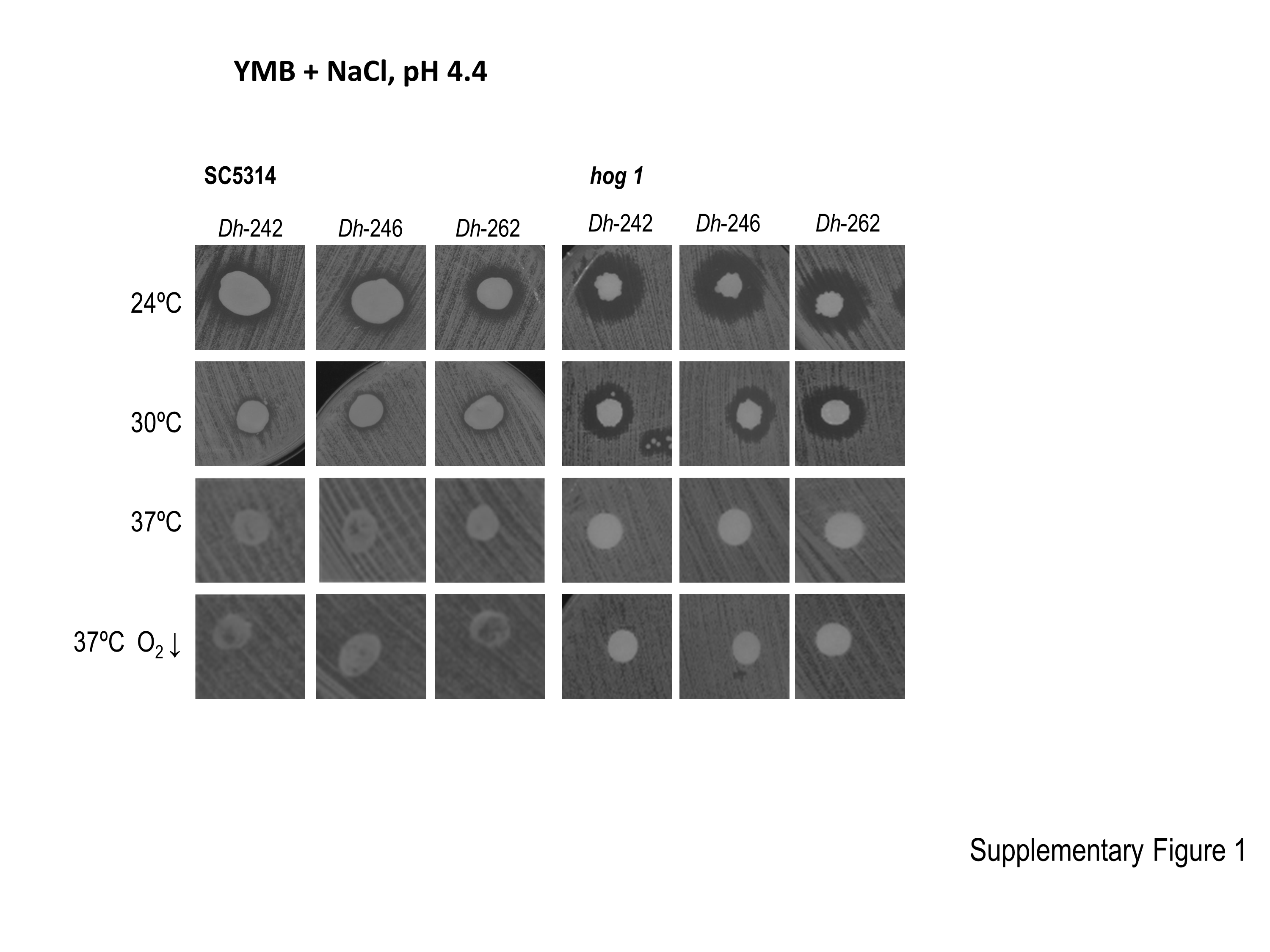

Supplement: Supplementary Figure 1 — Dh Kiler Toxin activity against C. albicans strains: The killer activity was analyzed on YMB plates supplemented with 3% NaCl pH 4.4 against SC5314 and the hog1 mutant strain under different environmental conditions. [file Image_1.TIF]

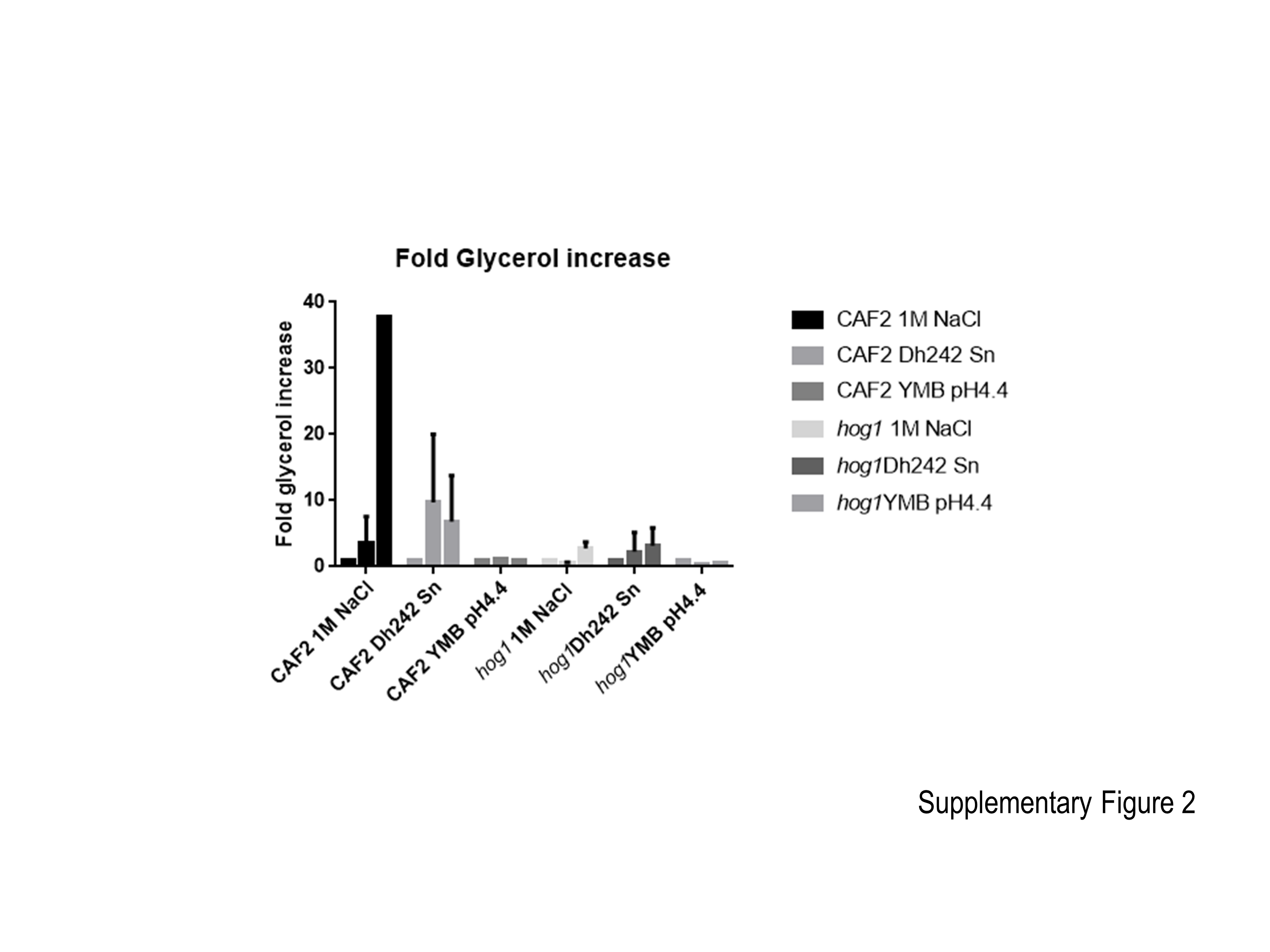

Supplement: Supplementary Figure 2 — Quantification of intracellular glycerol from CAF2 and hog1 mutant exposed to YMB plates plus 3% NaCl pH 4.4 (as control), supplemented with 1 M NaCl or Dh-242 supernatant at 0, 1 and 3 h. Fold increase is shown compared to glycerol level at time 0 for each strain. [file Image_2.TIF]

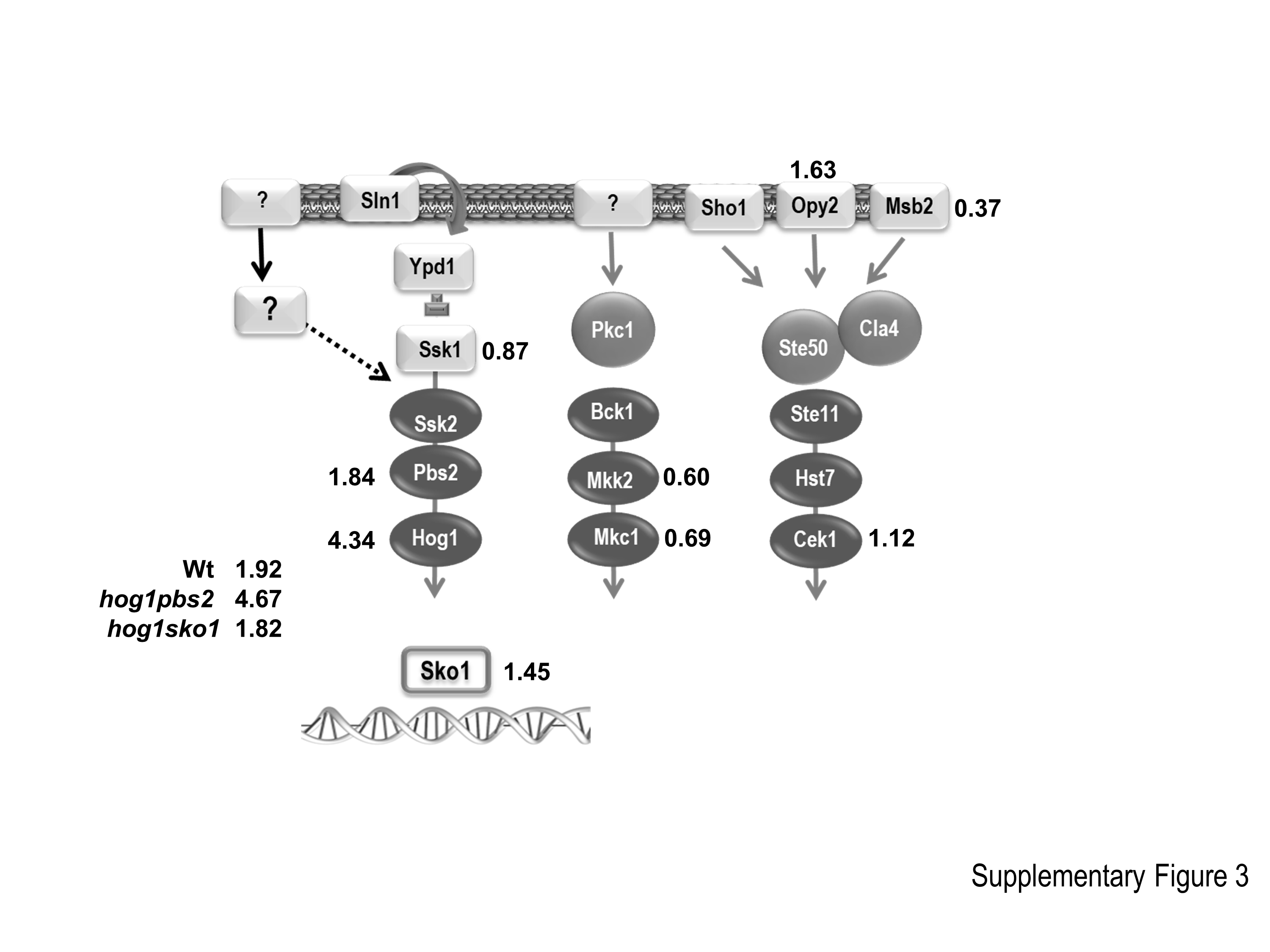

Supplement: Supplementary Figure 3 — Schematic graph of C. albicans MAPK signal transduction pathways analyzed in the present work. Transmembrane proteins and sensors are shown as rectangles, intermediate molecules are depicted as circles and MAP Kinase modules are ovals. The transcription factor Sko1 is shown as a white rectangle. Numbers indicate the inhibition zone (in mm) displayed by defective mutants in the specific elements in the presence of Dh-242 strain in YMB NaCl pH 4.4 at 30°C. [file Image_3.TIF]

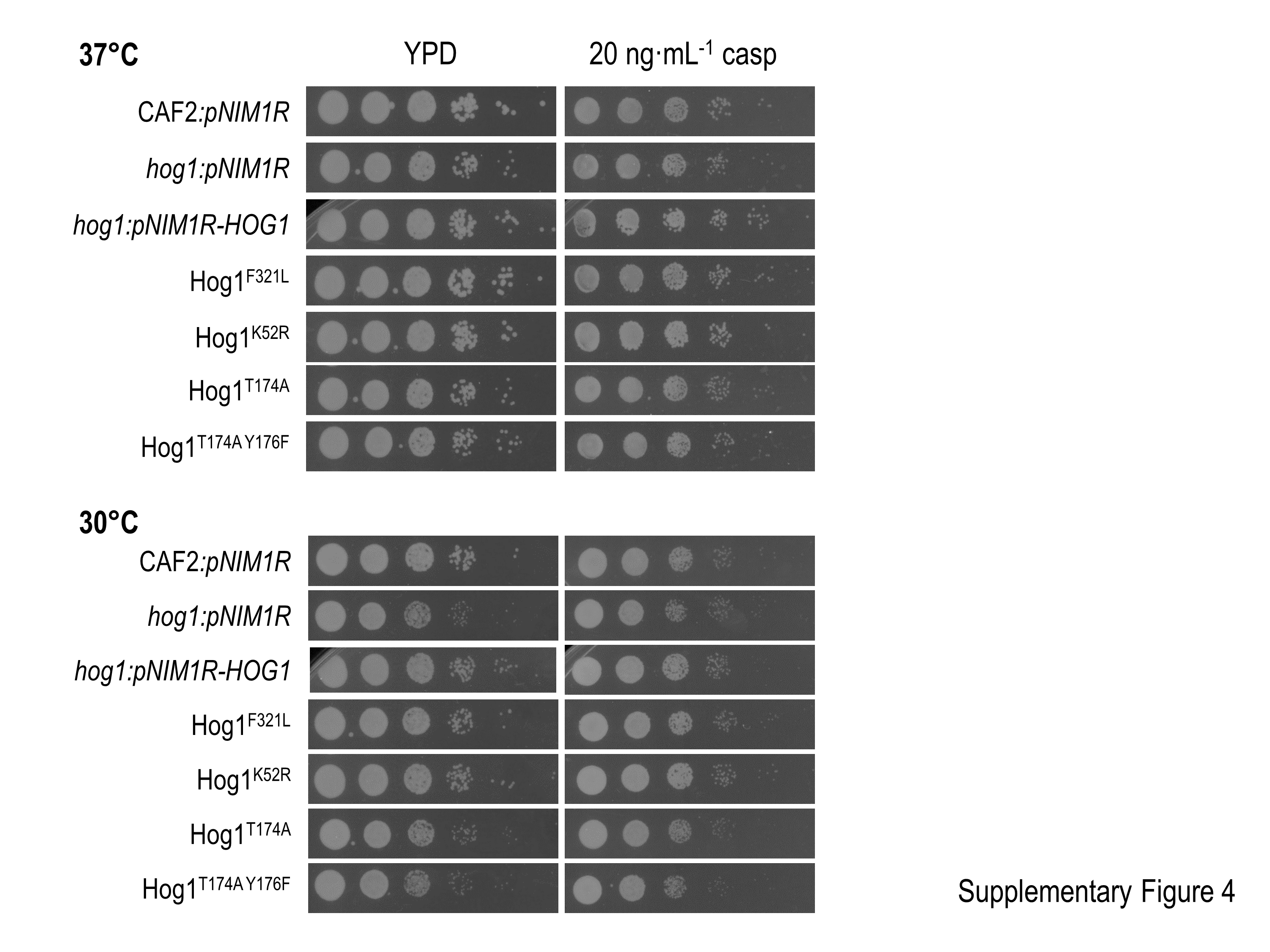

Supplement: Supplementary Figure 4 — Susceptibility of different strains to caspofungin. The indicated strains were spotted on YPD supplemented or not with caspofungin (Casp) and incubated at 30 or 37°C for 24 h before scanning. [file Image_4.TIF]

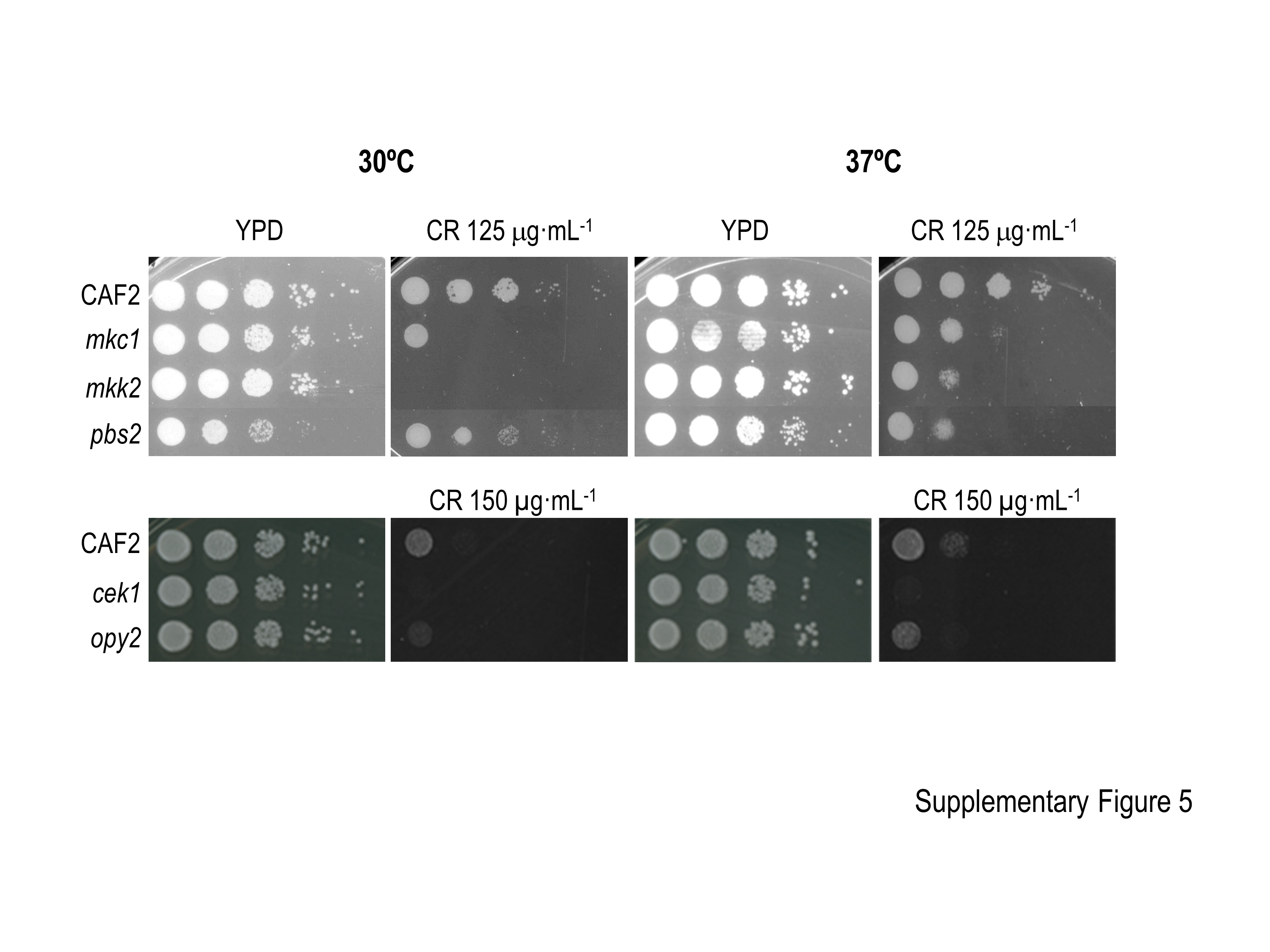

Supplement: Supplementary Figure 5 — The indicated strains were spotted on YPD supplemented or not with Congo red and incubated at 30 or 37°C for 24 h before scanning. [file Image_5.TIF]
